# Using Crop Databases to Explore Phenotypes: From QTL to Candidate Genes

**DOI:** 10.3390/plants10112494

**Published:** 2021-11-18

**Authors:** Anne V. Brown, David Grant, Rex T. Nelson

**Affiliations:** 1 United States Department of Agriculture-Agricultural Research Service, Corn Insects and Crop Genetics Research Unit, Ames, IA 50011, USA; anne.brown@usda.gov (A.V.B.); dgrant@iastate.edu (D.G.); 2 Department of Agronomy, Iowa State University, Ames, IA 50011, USA

**Keywords:** QTL, GWAS, candidate gene, genomics, genetics, database, SoyBase

## Abstract

Seeds, especially those of certain grasses and legumes, provide the majority of the protein and carbohydrates for much of the world’s population. Therefore, improvements in seed quality and yield are important drivers for the development of new crop varieties to feed a growing population. Quantitative Trait Loci (QTL) have been identified for many biologically interesting and agronomically important traits, including many seed quality traits. QTL can help explain the genetic architecture of the traits and can also be used to incorporate traits into new crop cultivars during breeding. Despite the important contributions that QTL have made to basic studies and plant breeding, knowing the exact gene(s) conditioning each QTL would greatly improve our ability to study the underlying genetics, biochemistry and regulatory networks. The data sets needed for identifying these genes are increasingly available and often housed in species- or clade-specific genetics and genomics databases. In this demonstration, we present a generalized walkthrough of how such databases can be used in these studies using SoyBase, the USDA soybean Genetics and Genomics Database, as an example.

## 1. Introduction

Since the introduction of bi-parental QTL analysis in plants [1] in the early 1980s, QTL regions have been described in both plant and animal species [2]. In early QTL analyses, the number of markers used and the limited number of progeny examined meant that the genetic regions encompassed by a QTL were usually large. These regions could include dozens, if not hundreds of genes, making candidate gene identification for the trait measured tedious, if not impossible (reviewed in [3]). Fine mapping with more markers is necessary to further limit the genetic region containing the gene conditioning the trait. This process would be aided if a naturally occurring or synthetic mutant in the gene conditioning the trait existed [4]. 

In previous years, fine-mapping was both a time consuming and expensive process that was not routinely performed to identify candidate genes. More recently, with the drop in sequencing costs, identification of vast numbers of single nucleotide polymorphisms (SNPs) and relatively inexpensive analysis technologies, it has become feasible to both identify smaller QTL regions and generate sequence information for those regions [5]. Additionally, Genome-Wide Association Studies (GWAS) utilizing SNP allele information have been employed to identify sequence regions associated with phenotypic traits and tools have been developed to integrate GWAS studies with QTL data such as QTLtools [6].

As more genomic data become easily accessible by quick and easy data sharing [7], some clade and species genome databases are now actively curating both bi-parental QTL and GWAS QTL information. This information can be used to identify candidate regions, although these regions typically contain many candidate genes. The list of candidate genes can often be reduced by considering molecular function annotations and tissue expression patterns. To illustrate this process, we will use, as an example, the information curated in the species database SoyBase [8]. 

SoyBase is the United States Department of Agriculture, Agricultural Research Service (USDA-ARS) soybean genetics and genomics database [8] and has been actively curated since its inception in the early 1990s. In 2010, the first assembly of a soybean genome (CV. Williams 82) was released [9]. Since then, SoyBase has been curating genomic information and presenting these data in the context of the original genetic data. We will demonstrate how genetic and genomic data can be used in silico to help identify candidate gene(s) that might condition a phenotype of interest. This process has often been referred to as phenotype to genotype (P2G) or field to genes (F2G).

## 2. Example Walkthrough

This demonstration on using a genomic/genetic database in P2G research was developed using SoyBase. Although the specific examples presented are for soybean, most species- or clade-specific databases will have somewhat equivalent data; however, the tools to display that data vary. In this demonstration, we present a series of steps that demonstrate how the various data types in SoyBase can be used together to identify a candidate gene controlling a trait. We do not intend to imply that the path through the database we present is the only one that would accomplish this, only that this is one way of solving the problem that highlights some of the important data sets available.

Seed oil is a major product extracted from soybeans, and seed oil composition is a significant factor in determining the price of oil paid by processors. Oil that contains reduced linolenic content is more stable during storage [10] and as a frying oil [11]. Thus, determining the genes and regulatory networks of linolenic synthesis is an important step in developing improved varieties, and this will be the trait used in this demonstration. The first step in identification of the gene(s) controlling seed linolenic acid content is to identify QTL for this trait, i.e., region(s) of the genetic map that have been associated with the phenotype. 

**Figure 1:**
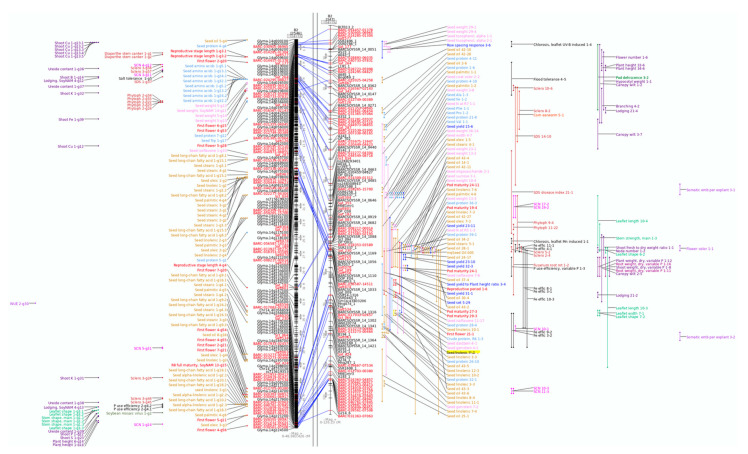
The composite genetic map and physical map of linkage group B2/chromosome 14. The left pane shows the physical or sequence map based on the soybean reference cultivar Williams 82. Genetically mapped molecular markers for which the sequence is available are shown on the physical map along with the gene models. The right pane shows the GmComposite2003 genetic map. This map was created in 2003 as the composite genetic map for soybean and is continually updated with new QTL and genetic markers. Markers in common between the two maps are connected by blue lines and shown in red text. Both bi-parental and GWAS QTL are grouped in columns by function or developmental category. Related QTL within categories are shown using the same color. Both QTL types use the same groupings and color to make correlations across the chromosome representations easier. The Seed linolenic 11-2 QTL is highlighted in yellow. Larger version.

In this example, we will use the SoyBase Search function to obtain a list of QTL for the search term “linolenic”. SoyBase contains information for 68 bi-parental QTL related to seed linolenic acid content that have been reported in 14 papers. Further examination of these results shows that there is a region on molecular linkage group B2 (chromosome 14) that has a large number of bi-parental QTL for seed oil traits, including several for seed linolenic acid content (Figure 1).

The SoyBase genetic map viewer is composed of two panes (Figure 1). The left shows a representation of the soybean physical or sequence map based on the Williams 82 genome sequence. This view of the chromosome shows the positions of molecular markers, the gene models (Glyma.14gxxxxxx) and the GWAS QTL identified in soybean. On the right is the soybean Composite Genetic Map, which shows the genetically mapped molecular markers along with the QTL identified in soybean. 

The hand-curated Composite Genetic Map is based on the reported QTL mapping studies in soybean and allows QTL from different publications to be displayed using a common coordinate system. Markers present on both the genetic and sequence maps are connected by a blue line. These two views of a chromosome allow the easy identification of regions with relatively high or low recombination as well as where the genetic and sequence maps are not congruent. In addition, comparing the locations of the bi-parental and GWAS QTL can provide information that is not available if used individually. Note that these two views of a chromosome have an important difference: coordinates on the sequence map are in base pairs (bp, left) while those on the genetic map are in centi-Morgans (cM, right).

**Figure 2:**
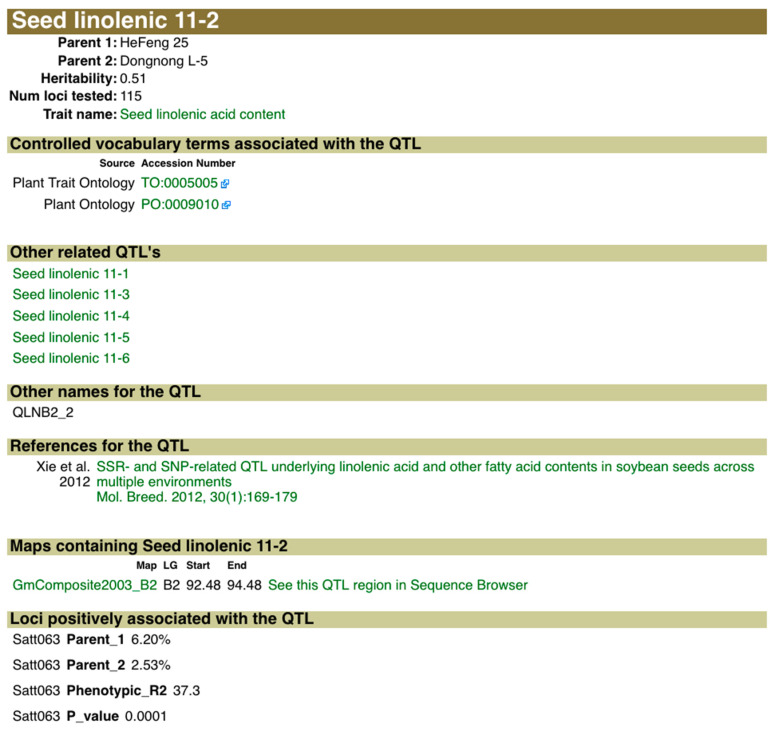
QTL report page for Seed linolenic 11-2. The QTL report for Seed linolenic 11-2 provides details on the QTL such as its heritability, parents and parental phenotype. It also lists any other phenotypes measured in the study (none in this example) and other QTLs for the trait identified in the study (Other Related QTLs). The map and location of the QTL is presented in the section “Maps containing Seed linolenic 11-2”. Clicking on the link “See this QTL region in Sequence Browser” will take the user to the sequence browser view of the approximate QTL on the sequence map to allow browsing of the gene model annotations. Genetic loci that are associated with the QTL are listed in the “Loci positively associated with the QTL” section along with association values for the loci.

**Figure 3:**
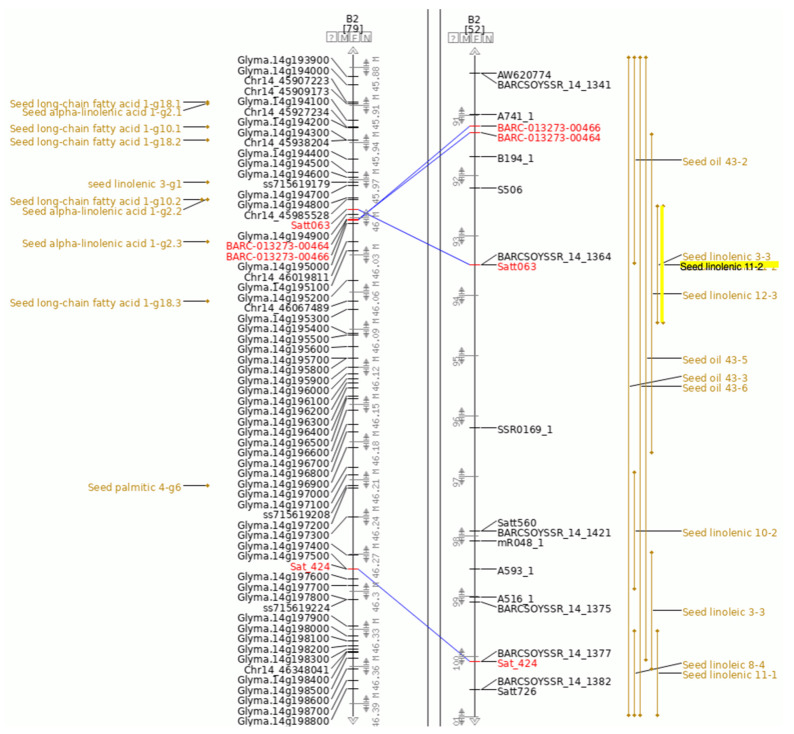
Genetic and physical map region containing Seed linolenic 11-2. Region of the physical and genetic map of MLG B2/Gm14 containing Seed linolenic 11-2. Only seed oil QTL are pictured for clarity. The physical map (**left**) includes the locations of gene models (Glyma.14g194300). The composite map (**right**) contains QTL regions for seed oil related QTL. Sequence based markers that have been genetically mapped are connected by blue lines. Larger version.

We will use Seed linolenic 11-2 as the QTL of interest in this example (Figure 2). Along with information about the cross used to identify this QTL and other related information, the QTL page for Seed linolenic 11-2 provides links to the QTL on the SoyBase Genetic Map and to the approximate region containing this QTL in the SoyBase Genome Sequence Browser. Seed linolenic 11-2 was originally identified as a bi-parental QTL where the inheritance of the trait was genetically associated with the molecular marker Satt063 (Figure 3). 

For clarity, in this example, only seed related QTL are shown. Comparison of the physical and genetic maps indicates that not only have there been many seed oil and linolenic content bi-parental QTL identified in the region but also that a number of GWAS QTL for seed oil content, linolenic acid and long-chain fatty acids are present in the corresponding region of the physical map. As this region contains many genes, a useful first step to identifying potential candidate genes is to view this region of the chromosome in the SoyBase Sequence Browser where a short annotation is provided for each gene. 

**Figure 4:**
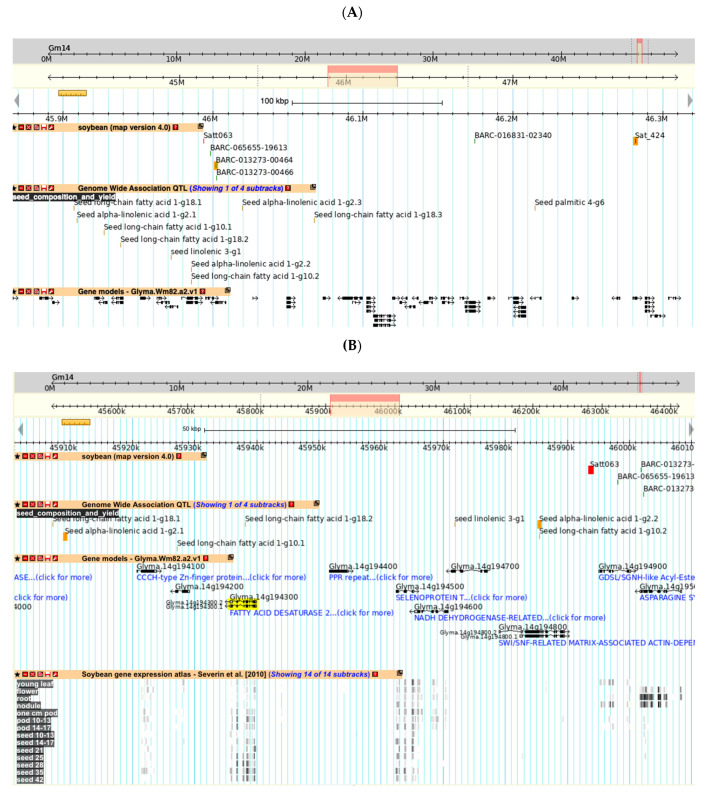
Identification of a candidate gene using the SoyBase Genome Browser. The region of the soybean physical map around Seed linolenic 11-2. (**A**) Magnification of the genomic region around Satt063. Molecular markers that flank Seed linolenic 11-2 are highlighted in orange. Tracks are also shown for GWAS QTL and genes. Larger version (**B**) Magnification of the chromosomal region in Panel A showing the short functional annotation for genes. The candidate gene Glyma.14g134300 is highlighted in yellow. The flanking GWAS QTL (orange) are indicated in the Genome Wide Association QTL track. Gene expression patterns indicating that the highlighted gene is preferentially expressed in seed tissue derived from RNA-seq are shown in the bottom track. Larger version.

This region can be viewed by selecting the closest flanking markers around the QTL (BARC-013273-00464 and Sat_424, shown in red text) and showing this region in the Sequence Browser (Figure 4A, flanking markers highlighted in orange). This figure also includes tracks for the related GWAS QTL and genes. Zooming into this view shows the short annotations for each gene (Figure 4B). In this view, a track showing gene expression as revealed by RNA-seq has been added.

Figure 4B shows several lines of evidence that point to Glyma.14g194300 (highlighted in yellow) as a candidate for the gene conditioning seed linolenic content:

- Located physically close to Satt063 (highlighted in red), the molecular marker most associated with Seed linolenic 11-2.
- Located within the region of GWAS QTL Seed alpha-linolenic acid 1-g2 (highlighted in orange).
- Annotated as a Fatty Acid Desaturase.
- Preferentially expressed in developing seeds.

**Figure 5:**
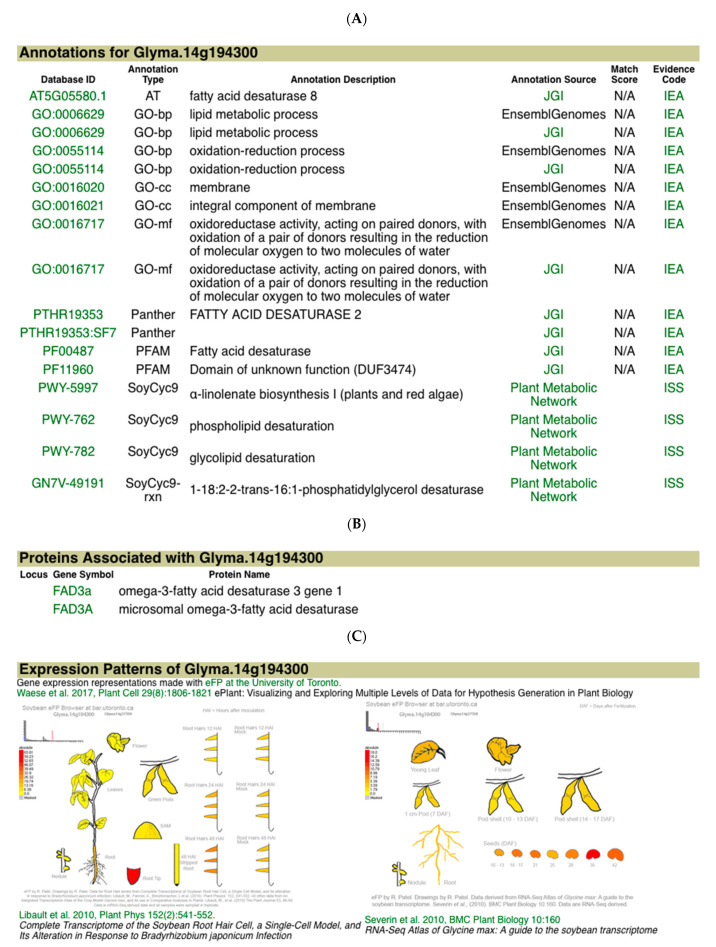
Detailed gene report for Glyma.14g194300. Details of the SoyBase gene report for the candidate gene Glyma.14g194300. (**A**) Functional and biochemical pathway annotation of the candidate indicates that it is a fatty acid desaturase and functions in the α-linolenate biosynthesis I pathway of plants and algae. Evidence codes are described at the GO evidence code page. (**B**) The protein product of this gene has been identified as FAD3A, a microsomal ω-3-fatty acid desaturase gene known to be involved in seed linolenic acid biosynthesis in soybean. (**C**) Expression of this gene measured by RNAseq is elevated in seed and shoot apical meristem tissue.

The information page for Glyma.14g194300 provides more information for this gene, parts of which are shown in Figure 5. Panel 5A gives the annotations from a number of sources for Glyma.14g194300. Panel 5B shows that the gene model is associated with the gene FAD3A, which is known to carry out a major step in linolenate biosynthesis and seed linolenic acid content [12]. Panel 5C presents a pictorial representation of the gene’s expression in different tissues and steps in development [13]. Glyma.14g194300 has relatively high expression during seed development, which supports the conclusion above that it is a candidate gene for the Seed linolenic 11-2 and Seed alpha-linolenic acid 1-g2 QTL.

In this example, there is a gene previously shown to be involved in the seed linolenic content phenotype. In cases where there is no obvious candidate gene in the region, other sources of information will be necessary to identify a strong candidate gene. Such supplementary information includes gene function (geneontology.org, accessed on 12 November 2021), protein structure (pfam.xfam.org, accessed on 12 November 2021), orthology (pantherdb.org, plants.ensembl.org, accessed on 12 November 2021), participation in biological pathways (plantreactome.gramene.org, plantcyc.org, accessed on 12 November 2021) and protein–protein interactions (string-db.org, accessed on 12 November 2021), which can be found in the respective databases.

**Figure 6:**
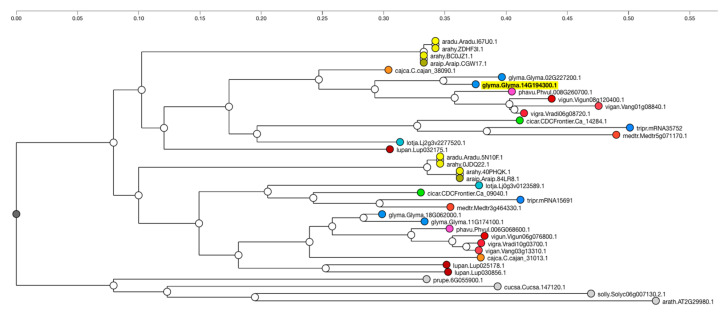
Orthologs of Glyma.14g194300. The phylogram derived from the Legume Information Service’s Phylotree viewer. Sequences with high sequence similarity to Glyma.14g194300 (highlighted in yellow) are from Common Bean (phavu), Cowpean (vigun), Adzuki Bean (vigan) and Mung Bean (vigra). Larger version.

Additionally, information regarding gene function can often be inferred from or to other species based on orthology or sequence similarity. Orthologs of Glyma.14g194300 in other species can identify genes that may also condition the seed linolenic content in those species. Orthologous genes in other species can be viewed by clicking the “View Gene Family’’ button on the Glyma.14g194300 report page. This will present a sequence similarity or ontology tree from the Legume Information System (LIS, legumeinfo.org, accessed on 12 November 2021) (Figure 6). It is often the case that other well-characterized species may appear in the tree. These can then be used as an additional source of information when inferring a candidate gene’s function.

As an extra set of conformation of QTL, a new tool called the Genotype Comparison Visualization Tool (GCViT) [14], available on Github (https://github.com/LegumeFederation/gcvit, accessed on 12 November 2021) and SoyBase, can be of use. GCViT is a tool that can be used with any species and will plot SNPs from multiple accessions and display where the differences in alleles are. Therefore, we can confirm/and or identify new regions for linolenic QTL by comparing lines with high linolenics to lines with low linolenics. Another tool that can be used to confirm QTL locations are ZBrowse [15] and ZZBrowse (https://zzbrowse.legumeinfo.org/, accessed on 12 November 2021) [16]. ZBrowse is an interactive tool for the visualization of GWAS data across experiments within a single species, while ZZBrowse is an interactive web tool for the comparative analysis of GWAS and QTL between species [16].

## 3. Conclusions

In this exercise, we demonstrated how a genetics/genomics database can be used as a tool to help identify the gene(s) conditioning a QTL. Although we used SoyBase in this exercise, other species- or clade-specific databases may contain equivalent data and tools that can be used in concert to accomplish a similar investigation. While other databases may collect similar data, they are not focused on the same user experience that SoyBase tools are. Thus, the path a user takes to identify candidate genes is unique to each database. 

A common theme of these databases is that they strive to collect what is known about a species’ genetics, genomics, phenotypes, biochemistry and other data into a single repository that allows users to quickly identify the information relevant to the question of interest. The reader will still have to consult some of the external databases referred to above and to other primary literature to manually identify candidate genes as no single species or clade database can assemble all relevant data for a single gene.

## Data Availability

All data on SoyBase.org (accessed on 9 November 2021) is publically available.
